# *Helicobacter cinaedi* bacteremia in a patient with primary central nervous system lymphoma

**DOI:** 10.1002/jha2.285

**Published:** 2021-09-16

**Authors:** Mai Fujita, Hiroshi Ureshino, Shinya Kimura

**Affiliations:** ^1^ Division of Hematology, Respiratory Medicine and Oncology Department of Internal Medicine Faculty of Medicine Saga University Saga Japan

A 61‐year‐old man diagnosed with primary central nervous system lymphoma (PCNSL) was admitted to our hospital for the second cycle of rituximab, methotrexate, procarbazine, and vincristine (R‐MPV) treatment. On day 6 of the second cycle of R‐MPV, erythema nodosum lesions emerged at his left leg and right wrist joint, and then disappeared after initiation of loxoprofen sodium hydrate. No fever was observed in the first or second cycle of R‐MPV. On day 1 of the third cycle of R‐MPV, erythema appeared on the left hip and left arm. The arm lesions disappeared rapidly after initiation of topical betamethasone, whereas the hip lesions remained. On day 5, the patient developed a high fever (39.2°C), and blood culture was performed. The patient did not suffer from neutropenia (white cell count, 5.2 × 10^9^/μl; neutrophils, 77.6%), diarrhea, or joint pain. After initiation of levofloxacin, fever was ameliorated within 24 h, but the erythema at the left hip was not ameliorated. Gram‐negative spiral bacilli were isolated (Figure 1A) by 5 day blood culture, and swarming colonies generated a thin film on Brucella agar (Figure 1B), indicating *Helicobacter cinaedi*. Subsequently, the bacteria were confirmed to be *H. cinaedi* by mass spectrometry. A 1‐week course of intravenous ampicillin was administered, followed by a 3‐week course of oral amoxicillin and a subsequent 3‐week course of oral kanamycin. After initiation of antibiotics, the erythema in the left hip disappeared rapidly without relapse.

**FIGURE 1:**
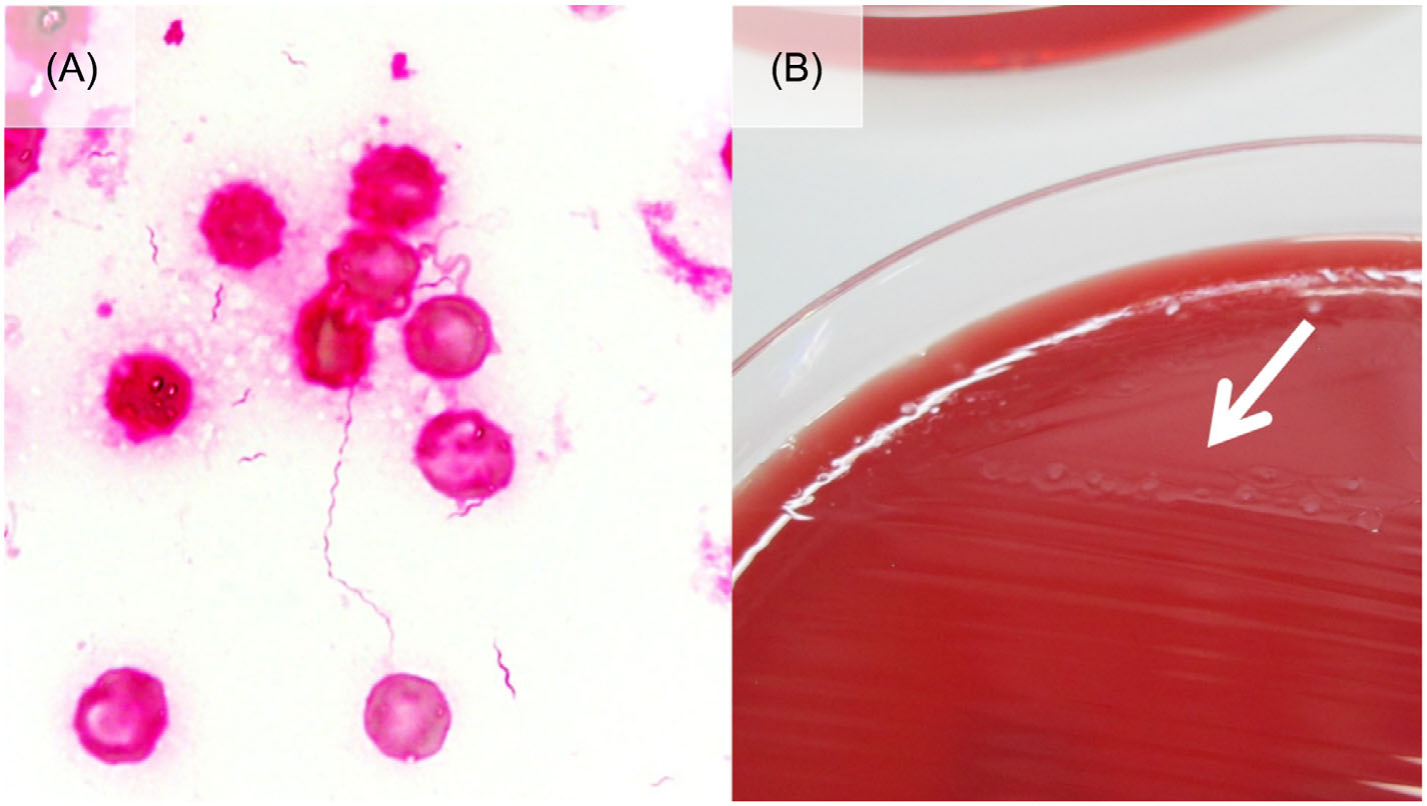
(A) A Gram‐negative spiral bacteria in blood culture (1000×). (B) Swarming colonies generated a thin film on Brucella agar after 5‐day incubation (white arrow)

*Helicobacter cinaedi*, a Gram‐negative spiral‐shaped bacterium that primarily resides in the gastrointestinal tract, can cause bacteremia accompanied by skin lesions, particularly in immunodeficient individuals. *Helicobacter cinaedi* is a slow‐growing bacteria that is difficult to detect by conventional blood culture due to the short incubation time of this procedure (>5‐day incubation time is required in half of cases). Accordingly, *H. cinaedi* may be an underdiagnosed cause of febrile illness. Physicians should consider the possibility of *H. cinaedi* bacteremia when patients with hematological malignancy complain of febrile illness with skin lesions. Spiral‐shaped Gram stain findings and thin film‐like colony formation on Brucella agar may help physicians to diagnose *H. cinaedi* infection.

## CONTRIBUTIONS

Mai Fujita, Hiroshi Ureshino, and Shinya Kimura were involved in the clinical care of the patient and manuscript preparation. Written informed consent for publication was obtained.

## CONFLICT OF INTEREST

The authors declare no potential conflict of interest.

## DATA SHARING

All data can be obtained by a reasonable request from the corresponding author.

